# Supplementary material for: Purine Auxotrophic Starvation Evokes Phenotype Similar to Stationary Phase Cells in Budding Yeast
Source: J Fungi (Basel). 2021 Dec 29;8(1):29. doi: 10.3390/jof8010029 (PMC8780165; doi:10.3390/jof8010029)
Supplement: Supplementary file 1 [file jof-08-00029-s001.zip › Supplementary File S1.pdf]

	CO_2	ethanol	acetate	glycerol	biomass	glucose uptake	Specific growth rate μ [h^{-1}]
SD+ 1	32,5926	42,5513	1,21479	1,51756	17,0405	86,5755	0,39875
SD+ 2	24,8146	34,5129	1,86647	1,22979	19,2025	75,6468	0,44934
SD+ 3	31,2419	31,4441	1,64147	0,92436	16,7734	81,3465	0,3925
Ade-1	9,15992	16,2818	0,63756	2,86901	6,58208	43,0184	0,15402
Ade-2	9,76494	16,0706	1,25228	3,71468	4,89126	40,5307	0,11446
Ade-3	4,11028	21,9096	0,98627	4,14574	7,55704	47,6182	0,17683

Specific production (or consumption in glucose case) q values in mCmols $*gDW^{-1} * h^{-1}$ from *ade8* cultivations in SD+ and ade- medias. Cultivations were performed in triplicates in 0.4 L reactors (starting volume 0.3 L). Samples from SD were taken after 4 hours of cultivation when max specific growth rate was reached. Samples from starving cells were harvested after 5.5 h of cultivation.
