# Supplementary material for: Purine Auxotrophic Starvation Evokes Phenotype Similar to Stationary Phase Cells in Budding Yeast
Source: J Fungi (Basel). 2021 Dec 29;8(1):29. doi: 10.3390/jof8010029 (PMC8780165; doi:10.3390/jof8010029)
Supplement: Supplementary file 1 [file jof-08-00029-s001.zip › Supplementary File S4.pdf]

NGS data analysis

Sequencing reads were quality filtered (Q=30), Illumina adapters and poly-A tails were removed and reads with a length of at least 100 nt were selected for further processing using cutadapt:

```
cutadapt -j 4 -a GATCGGAAGAGCACACGTCTGAACTCCAGTCAC -a A{100} -q 30,30 -m 100  -o out_file.format in_file.format
```

Reads were aligned using STAR aligner:

```
STAR --runThreadN 4 \
--genomeDir /c/star-genome \
--sjdbGTFfile /c/Saccharomyces_cerevisiae.R64-1-1.96.gtf \
--outFilterType BySJout \
--outFilterMultimapNmax 20 \
--alignSJoverhangMin 8 \
--alignSJDBoverhangMin 1 \
--outFilterMismatchNmax 999 \
--outFilterMismatchNoverLmax 0.04 \
--alignIntronMin 20 \
--alignIntronMax 1000000 \
--alignMatesGapMax 1000000 \
--quantMode GeneCounts \
--outSAMtype BAM SortedByCoordinate \
--limitBAMsortRAM 1287043197 \
--readFilesIn /mnt/e/liepins/liepins-ngs/53-samples-all/fg1/cut*fastq
```

Aligned reads were counted using HTSeq:

```
htseq-count -m intersection-strict -s yes -f bam -r pos Aligned.sortedByCoord.out.bam \
/mnt/h/ngs-liepins/20190424_214158/Fastq/gtf-sc/Saccharomyces_cerevisiae.R64-1-1.96.gtf \
> /c/counts.txt
```

edgeR was used for differential expression comparisons. Genes with less than 1 count per million (CPM) in less than 2 samples were filtered out, Benjamini and Hochberg method was used to calculate multiple comparison adjusted p-value as false discovery rate (FDR). FDR < 0.001 and logFC > 2 was set as a threshold for significance.

Workflow after adapter removal is also available on Galaxy Europe: https://usegalaxy.eu/u/karlispleiko/w/rna-seq-kp-single-read-after-cutadapt
